# Discoveries Interview: Doctor John D. Halamka on the digital healthcare revolution

**DOI:** 10.15190/d.2018.5

**Published:** 2018-07-11

**Figure 1:**
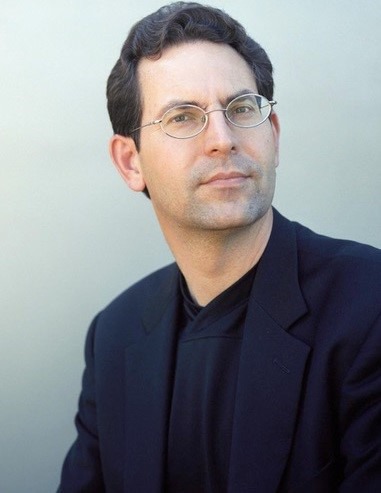
Doctor John D. Halamka

**Doctor John D. Halamka** MD, MS, is the chief information officer (CIO) at Beth Israel Deaconess System, chairman of the New England Healthcare Exchange Network (NEHEN), and a practicing emergency physician. He is the International Healthcare Innovation prof. at Harvard Medical School.

Dr. Halamka completed his undergraduate studies at Stanford University, where he received a degree in medical microbiology and a degree in public policy with a focus on technology issues. He entered medical school at the University of California, San Francisco and simultaneously pursued graduate work in bioengineering at the University of California, Berkeley focusing on technology issues in medicine. He completed his residency at Harbor–UCLA Medical Center in the Department of Emergency Medicine. While at Harbor-UCLA he developed a hospital-wide knowledge base for policies, procedures, and protocols. Moreover, he played an important role in the development of an online medical record, a quality control system, and several systems for medical education.

In 1996, Dr. Halamka joined the faculty of the Harvard Medical School, integrating his knowledge of medicine and technology. In his role at BIDMC, Dr. Halamka is responsible for all clinical, financial, administrative, and academic information technology, serving 3,000 doctors, 12,000 employees, and 1,000,000 patients. As chairman of NEHEN, Dr. Halamka oversees clinical and administrative data exchange among the payers, providers, and patients in Massachusetts. As a Harvard professor, he has served the George W. Bush admin-istration, the Obama administration, and national governments throughout the world planning their healthcare IT strategy.

Dr. Halamka has authored five books on technology-related issues, hundreds of articles and thousands of posts on the popular Geekdoctor blog.

He runs Unity Farm in Sherborn, MA and serves as caretaker for 150 animals, 30 acres of agricultural production and a cidery/winery.

## 1. The healthcare industry is entering the digital age. Can you describe in simple terms what is the digital healthcare technology and how it will transform the healthcare?

Digital healthcare is more than just making existing paper workflows electronic - it’s about entirely redefining the way healthcare is delivered now that digital tools are available. Here are a few examples.

Five years ago, my father in law began speaking in “word salad” - the sentences coming from his mouth were out of sync with the thoughts coming from his brain. My wife immediately called me and asked what to do. I suggested that she take him to the nearest community hospital with a CT scanner and telestroke capability, about 15 minutes away. Instead of traveling to an urban setting with nationally known stroke experts, my father in law received the necessary diagnostic studies in a rural environment, then connected with a neurology expert virtually from the bedside. The neurologist reviewed the CT images, patient history, and treatment options. With medication, the stroke cleared rapidly and my father in law was discharged the following day, completely back to baseline.

A year ago, my wife noticed her heart rate was elevated and she had an unplanned weight loss. Her hair was brittle and her eyebrow hair was thinning. These are the hallmarks of thyroid disease. Instead of driving into Boston, paying $40 for parking, and waiting for an overburdened primary care physician, my wife did a telecare visit, arranged for labs to be drawn at a nearby rural testing center, and received a referral to an endocrinologist. She did have an in person visit with the thyroid specialist and received medication (e-prescribed via an app) which resolved all her symptoms. Primary care, specialty care, pharmacy and lab were all coordinated with smart phones, cloud services and apps.

Finally, six months ago I was diagnosed with primary hypertension. My clinician asked me to use internet of things devices in my home (all consumer devices from Withings that cost less than $100) to monitor my blood pressure during activities of daily living. That data was sent to my phone and from my phone to the clinician's electronic health record with my consent. Based on lab tests done at a rural testing center and data from my home, he was able to develop a treatment plan and electronically prescribed medication. We continued telecare interactions to rapidly titer my dose of medication to personalize it to me.

## 2. How the healthcare technology field evolved over the time?

In the early 2000’s only 10% of hospitals and 10% of clinicians had fully electronic workflows. Today it’s greater than 90%. That means the pace of innovation, the need for increased reliability, and the security considerations have changed remarkably over the past decade.

## 3. How the Telemedicine and Internet of Things Technologies will impact healthcare?

The CHRONIC Care Act was passed and signed into law in February 2018 as part of the Bipartisan Budget Act of 2018. It relaxes geographic and facility-type restrictions under Medicare fee for service for telestroke services and adds the patient’s home and independent renal dialysis facilities as originating sites for telehealth dialysis services. The CHRONIC Care Act also gives Medicare Advantage plans the ability to offer telehealth services as part of their basic benefit package. For certain accountable care organizations (ACOs), the Act eliminates rural geographic area requirements, and enables homes to qualify as originating sites.

## 4. What will the field look like in 5-10 years? There are 5 trends to watch:

a. Work by providers and patients will be done on mobile devices. The smartphone is the platform of the future.

b. Increasingly, software and data storage is cloud based. The era of locally installed operated software is ending.

c. Homes will be filled with devices that measure healthcare parameters and monitor our wellness. Staying healthy in the home will be more important than visiting a hospital when you’re sick.

d. The genome and advances in research will make medicine more tailored to the individual - precision medicine.

e. Patients and families will have better tools to navigate the healthcare system, becoming equal partners in their care.

## 5. What advice do you have for young scientists and doctors?

The nature of work is changing entirely. Remain agile and accept that every day will offer you new opportunities. Be bold!

